# Heterotaxy Syndrome: Discordant Growth

**DOI:** 10.7759/cureus.14766

**Published:** 2021-04-30

**Authors:** Pratiksha Yadav, Pranav Ajmera, Sruthi Krishnamurthy, Nikhil B Nandivada

**Affiliations:** 1 Radiology, Dr. D.Y. Patil Medical College, Hospital and Research Centre, Pune, IND

**Keywords:** heterotaxy, polysplenia, partial anomalous pulmonary circulation, cardiac defects, situs ambiguous

## Abstract

Heterotaxy syndrome implies a discordance between placement of thoracic organs with respect to abdominal organs. A large number of these have associated congenital heart defects. This syndrome is unique as every patient is different and can have any permutation and combination of symptoms. In our case, the five-year-old male child presented with complaints of abdominal distension, fever, and bluish discoloration of limbs with even mild exertion. Radiological evaluation was diagnosed with a large atrial septal defect, cardiomegaly, partial pulmonary venous circulation, multiple small spleens on the right side of body, a large midline liver, malrotated bowel, inferiorly displaced kidneys, and two hemiazygos veins. The echocardiography and electrocardiogram too were consistent with atrial septal defect and right ventricular strain pattern.

The reasons for this highly variable pattern are rooted in the genetically complicated process of lateralization with a strong link to the copy number variations. Due to the variable patterns, it is more efficient to report all the findings utilizing a step-by-step process of commenting on each and every individual organ, instead of classifying them under different categories based on atrial isomerism. This is important as any other way of classification predisposes to a certain bias.

## Introduction

Heterotaxy syndrome, also known as situs ambiguous, is a rare abnormality with some figures stating their existence to be less than one per 10,000 [1]. The term situs in congenital heart diseases refers to the position of the chambers of the heart and visceral organs with respect to the midline. While situs solitus implies normal position of all the structures, situs inversus implies placement of all the structures in a mirrored fashion, and situs ambiguous implies a discordance between the placement of the thoraco-abdominal organs compared to the midline [2,3]. In over 50% of cases, heterotaxy has been found to be associated with congenital heart diseases. This syndrome is unique as every patient is different and can have any permutation and combination of symptoms. Due to the variable patterns, it is more efficient to report all the findings utilizing a step-by-step process of commenting on each and every individual organ, instead of classifying them under different categories based on atrial isomerism. This is important as any other way of classification predisposes to a certain bias.

## Case presentation

A five-year-old male child presented to the pediatric department with complaints of gradual abdominal distension developing since few months as well as fever and bluish discoloration of the limbs since few days. The mother complained that her son got tired easily on exerting himself during activities such as playing.

Under the routine admission protocol, the entire panel of blood markers was sent, electrocardiogram was performed, and the patient was then sent to the radiology department for an ultrasonographic evaluation and radiography. The hemoglobin was 9 gm/dL, and the packed cell volume (PCV), mean corpuscular volume (MCV), and mean corpuscular hemoglobin concentration (MCHC) too were below the lower limit of normal. The total leucocyte count at 23,900/μL was significantly raised, and differential leucocyte count revealed eosinophils at 60%. The electrocardiogram revealed a right bundle branch block with right ventricular strain pattern.

**Figure 1:**
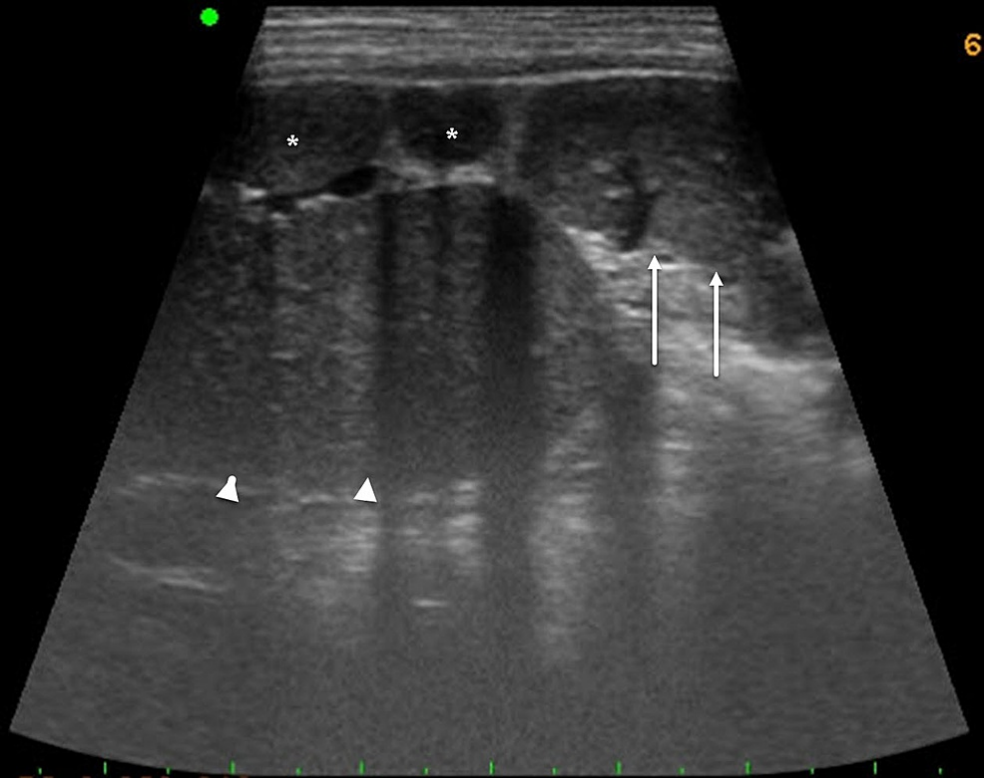
Oblique sections on gray-scale sonography of a five-year-old child suffering from heterotaxy syndrome acquired over the left upper abdomen, slightly posteriorly, revealing multiple spleens (asterisk), extension of enlarged right lobe of liver (arrowheads), and a malpositioned inferiorly displaced left kidney (arrows).

Ultrasonography (USG) of abdomen and pelvis revealed an enlarged midline liver with two sets of dilated hepatic veins; multiple splenules were seen on the right side with empty left splenic region. Due to the enlarged liver, the right kidney was noted at a significantly lower position with respect to the left kidney (Figure 1). There was intestinal malrotation with stomach located on the right side and small bowel loops clustered together on the left side; large bowel loops were clustered together on the right side; and sigmoid colon is noted in the lower half of right-side abdomen.

**Figure 2:**
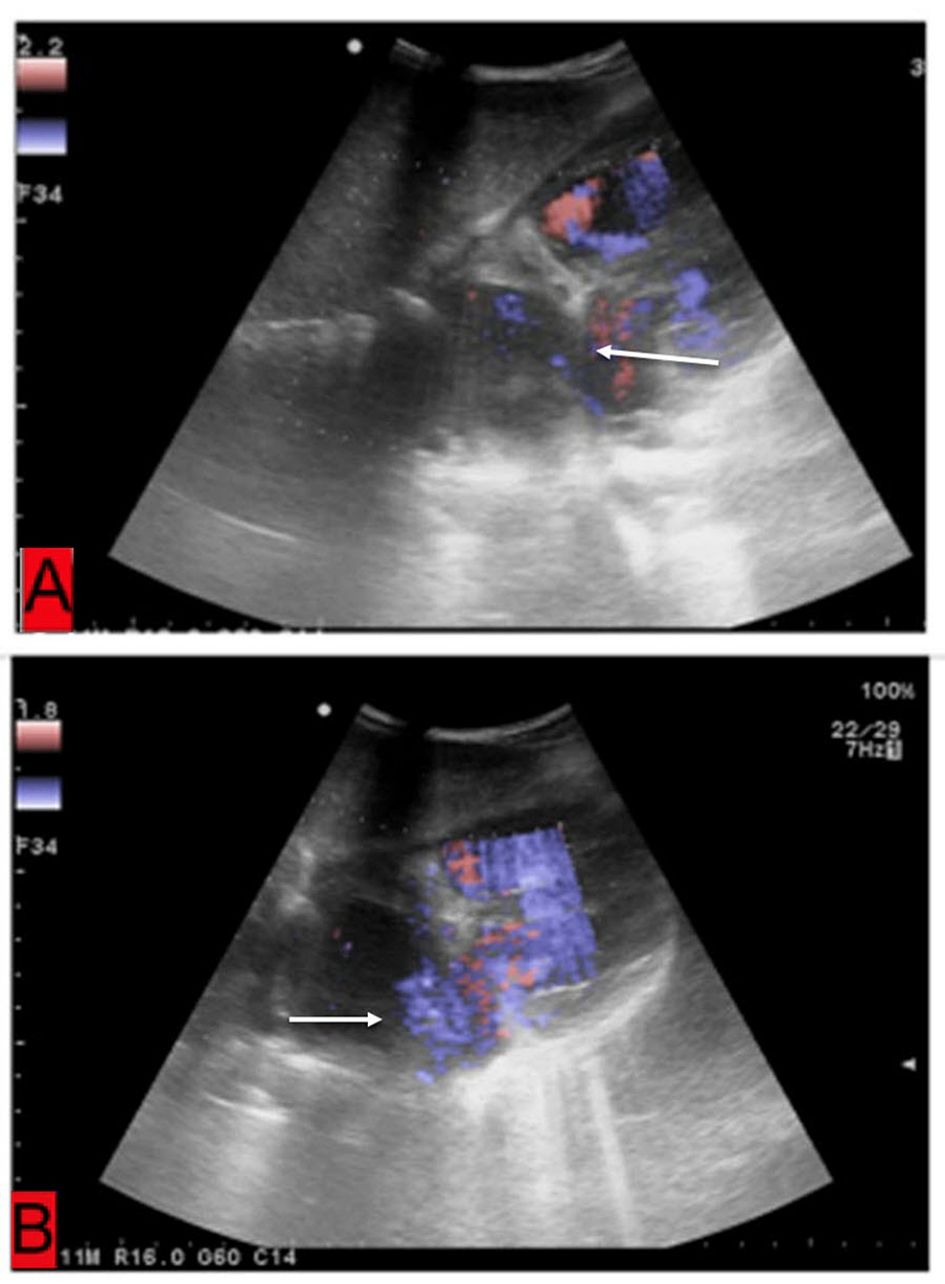
(A and B) Gray-scale sonographic visualization of heart of a five-year-old child suffering from heterotaxy syndrome revealing a large defect in the inter-atrial septum.

The radiograph revealed cardiomegaly with a cardiothoracic ratio of 0.7. In view of the above findings, an urgent two-dimensional echocardiography was performed, which revealed a severely dilated right atrium at the right ventricle with a moderate ostium secundum and bidirectional atrial septal defect; patent ductus arteriosus and severe tricuspid regurgitation and pulmonary artery hypertension too were observed (Figures 2A, 2B).

**Figure 3:**
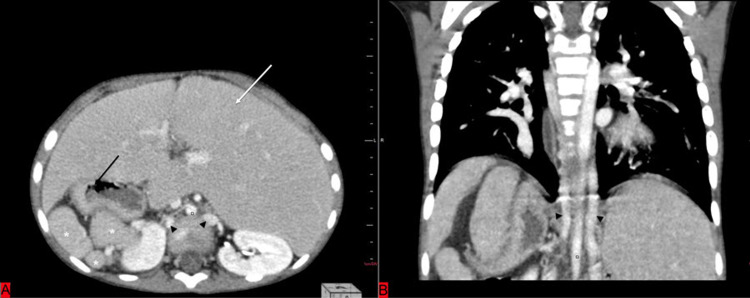
Reconstructed axial (A) and coronal (B) sections of abdominal CT of a five-year-old child suffering from heterotaxy syndrome revealing an enlarged liver (white arrow), multiple small-sized spleens (asterisk), fundus of stomach present on right side (black arrow), a malpositioned right kidney, and two hemiazygos veins (black arrowheads).

The CT revealed visceral situs inversus with multiple splenules seen in the right hypochondrium and an enlarged midline liver (Figures 3A, 3B).

**Figure 4:**
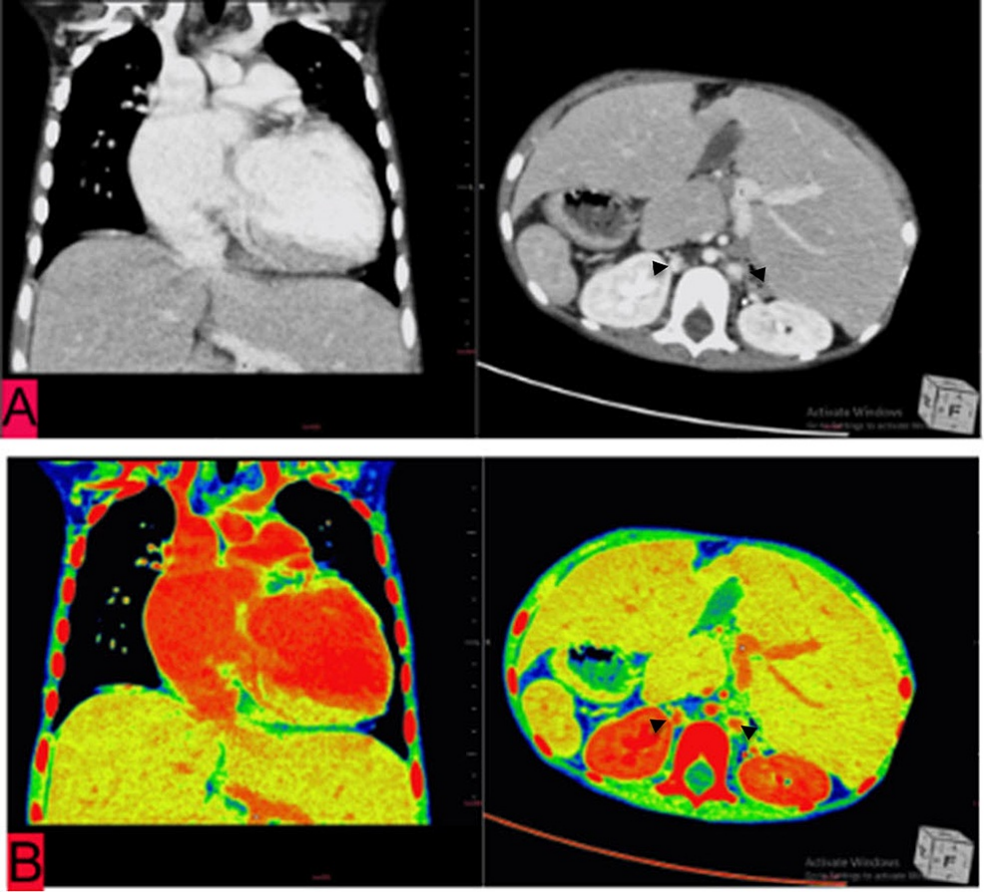
Axial and coronal sections of chest CT of a five-year-old child suffering from heterotaxy syndrome reconstructed on abdominal window (HU) (A) and then on PET-CT window (B) for better visualization revealing a central aorta flanked by a hemiazygos vein on either side (black arrowheads), which also reveals the left branch of portal vein PET-CT, Positron emission tomography–computed tomography.

Two veins in the abdomen were visualized, one each to the right and left side of the aorta; these were hemiazygos veins that join superior to the liver, and later this joins the azygous vein and then into the right atrium (Figures 3B, 4A, 4B).

**Figure 5:**
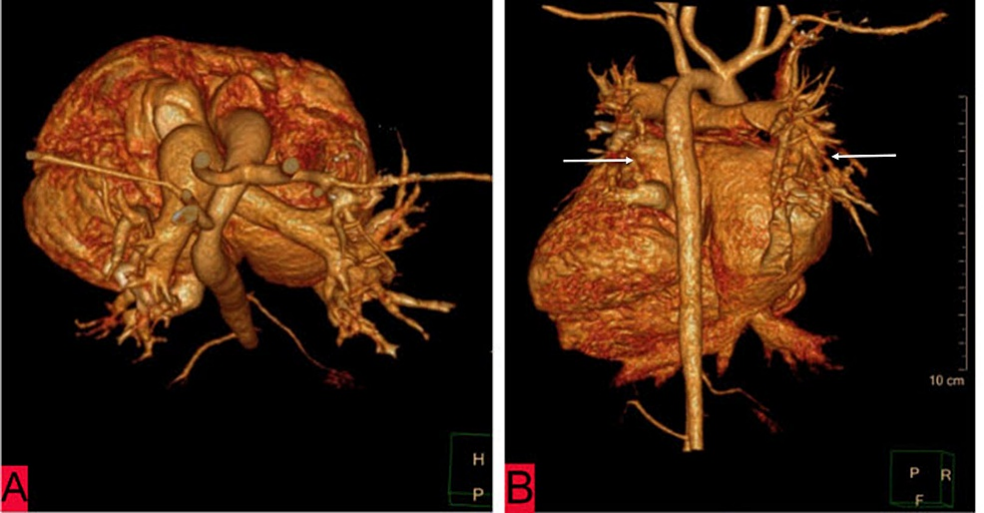
Reconstructed volume-rendered angiographic sections of chest CT of a five-year-old child suffering from heterotaxy syndrome revealing malposition of great vessels (A) and a veno-arterial discordance where the right-sided superior and inferior pulmonary veins were seen joining the right atrium, while the left-sided superior and inferior pulmonary veins were seen draining into the left atrium (B).

It revealed an ambiguous thoraco-abdominal situs with two lobes and two bronchi in both the lungs; dextro-malposition of the great vessels with the aorta seen on right side of pulmonary artery but with normal atrio-ventricular concordance, and D-loop configuration of the ventricle. There was a veno-arterial discordance, and the right-side superior and inferior pulmonary veins were seen joining the right atrium, while the left-side superior and inferior pulmonary veins were seen draining into the left atrium (Figures 5A, 5B).

**Figure 6:**
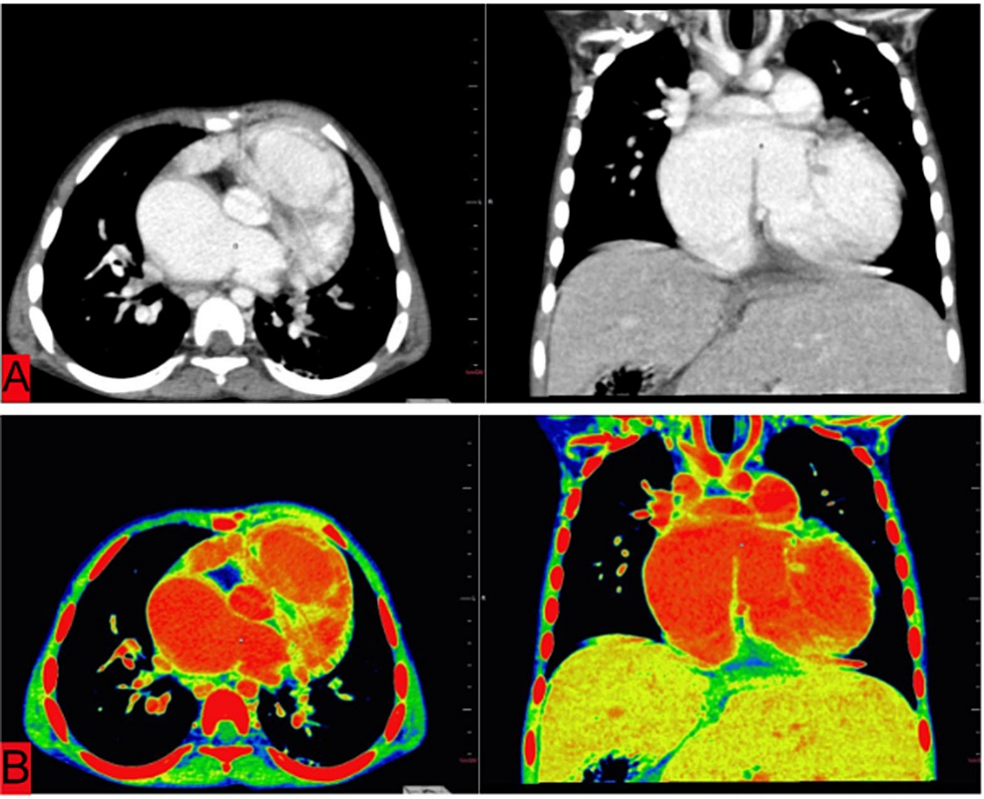
Axial and coronal sections of chest CT of a five-year-old child suffering from heterotaxy syndrome reconstructed on abdominal window (HU) (A) and then on PET-CT window (B) for better visualization revealing a large inter-atrial septal defect (small square), measuring nearly 1.4 cm. PET-CT, Positron emission tomography–computed tomography.

Hence, the patient had a partial anomalous pulmonary venous circulation. The inter-atrial septum showed a large defect of size nearly 1.4 cm, and the overall heart was enlarged in size (Figures 6A, 6B).

## Discussion

While the existing classification classifies situs ambiguous either with respect to the presence of the spleen or with respect to the atrium, these are in reality only associations [4]. Each case can have virtually any possible combination of abnormalities, making each case unique [5]. However, due to the multiple abnormalities, the prognosis is usually poor for such patients if not treated in time, and most of them do not survive beyond one year of age. However, this was an atypical case of heterotaxy in which the patient was asymptomatic till five years of age and was developing normally for children of his age.

The reason for the highly variable presentation of heterotaxy lies in mutations in the genetically complicated process of lateralization, with also a strong link between the copy number variation genes and phenotypic expression. An idiosyncrasy in the predetermined formation of the second heart field structures explains why some heart defects present more frequently than others [1,5,6].

**Figure 7:**
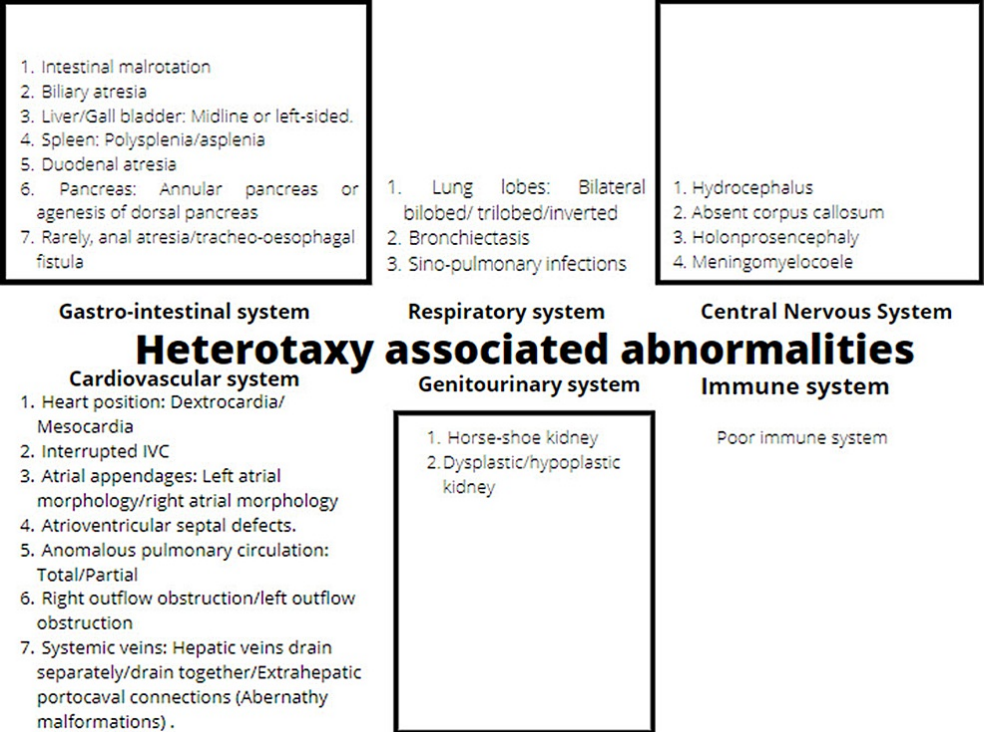
A diagrammatic representation of the various possible manifestations that have been documented in cases of heterotaxy.

These mutations are the reason that each case needs to be documented systematically and in a stepwise manner as there is no 100 percent association between abnormalities of two organs, which occur mostly independent of each other. One systemic abnormality should not be used to predict the occurrence of another, and they all should be excluded separately [3,7,4]. The system-wise manifestations that can occur in a case of heterotaxy have been summed up together in the flow chart shown in Figure 7 [6,8-10].

Among all these systems, the cardiovascular system is most commonly involved, and the patient is generally present with a cardiac symptom. In our case too, the patient presented due to acute circulatory problems, and the abdominal findings were diagnosed as part of the systematic work-up.

The authors are of the view that the individualized approach as proposed by Applegate et al. is useful in ensuring that every organ has explained in relation to each other and in sufficient detail [2].

Our patient had polysplenia, and since both asplenia and polysplenia are predisposed to the chances of infection, it is important to be more cautious. Therefore, the parents were explained as such and counseled to come at the earliest onset of even minor infectious symptoms [10].

## Conclusions

In conclusion, this disorder is one of the greatest challenges faced by pediatric cardiologists and gastroenterologists to plan and treat due to the highly variable presentation. The accurate systematic radiological description of every organ is extremely valuable for predicting the possible future complications the child may face and hence to take pre-emptive steps when necessary.
